# Resistance mechanisms and population structure of highly drug resistant *Klebsiella* in Pakistan during the introduction of the carbapenemase NDM-1

**DOI:** 10.1038/s41598-019-38943-7

**Published:** 2019-02-20

**Authors:** Eva Heinz, Hasan Ejaz, Josefin Bartholdson Scott, Nancy Wang, Shruti Gujaran, Derek Pickard, Jonathan Wilksch, Hanwei Cao, Ikram-ul Haq, Gordon Dougan, Richard A. Strugnell

**Affiliations:** 1 Parasites and Microbes, Wellcome Trust Sanger Institute, Hinxton, CB10 1SA UK; 2 Department of Clinical Laboratory Sciences, CAMS, Jouf University, Al-Jouf, Saudi Arabia; 3 Department of Microbiology and Immunology, The University of Melbourne, at Peter Doherty Institute for Infection and Immunity, Melbourne, Australia; 4 Department of Microbiology, The Children’s Hospital & The Institute of Child Health, Lahore, Pakistan; 5 Department of Medicine, University of Cambridge, Cambridge, UK; 6 Institute of Industrial Biotechnology, GC University, Lahore, Pakistan

## Abstract

*Klebsiella pneumoniae* is a major threat to public health with the emergence of isolates resistant to most, if not all, useful antibiotics. We present an in-depth analysis of 178 extended-spectrum beta-lactamase (ESBL)-producing *K. pneumoniae* collected from patients resident in a region of Pakistan, during the period 2010–2012, when the now globally-distributed carbapenemase *bla*-NDM-1 was being acquired by *Klebsiella*. We observed two dominant lineages, but neither the overall resistance profile nor virulence-associated factors, explain their evolutionary success. Phenotypic analysis of resistance shows few differences between the acquisition of resistance genes and the phenotypic resistance profile, including beta-lactam antibiotics that were used to treat ESBL-positive strains. Resistance against these drugs could be explained by inhibitor-resistant beta-lactamase enzymes, carbapenemases or *ampC* type beta-lactamases, at least one of which was detected in most, but not all relevant strains analysed. Complete genomes for six selected strains are reported, these provide detailed insights into the mobile elements present in these isolates during the initial spread of NDM-1. The unexplained success of some lineages within this pool of highly resistant strains, and the discontinuity between phenotypic resistance and genotype at the macro level, indicate that intrinsic mechanisms contribute to competitive advantage and/or resistance.

## Introduction

Coincident with the development of antibiotic therapy, there has been a steady increase in the numbers and types of bacterial pathogens acquiring resistance to antimicrobials, with some clades showing a propensity to spread globally^1,2^. *Klebsiella pneumoniae* has been particularly successful in this regard and is a member of the ESKAPE pathogen group that are acknowledged as causes of serious infections associated with multidrug resistance^3,4^. Before this rapid increase in drug resistance, *K. pneumoniae* was primarily known as a major cause of infections in neonates, especially in Low- and Middle-Income Countries (LMICs)^5–8^ and community-acquired and nosocomial infections in immunocompromised patients^9–11^.

The population structure of *K. pneumoniae* was redefined recently as a species ‘complex’. This complex includes *K. pneumoniae sensu stricto*, which poses the main burden of hospital-acquired infections; *K. variicola*, which can be plant-associated^12^ but also causes hospital infections^13–15^; and *K. quasipneumoniae*, which were initially thought to be asymptomatic carriage isolates, until more recent reports highlighted their potential virulence and increased drug resistance^16–18^. DNA sequence-based phylogenetic typing analysis of the *K. pneumoniae* species has revealed complex, deeply branched lineages, and these data can be used to inform the epidemiological analysis of different high-risk, globally distributed clones^19^.

The acquisition of extended-spectrum beta-lactamases (ESBLs) rapidly increased in *K. pneumoniae* from the 1990s, particularly in hospital isolates^20,21^, and was driven by mobile elements (usually plasmids) often encoding several other resistance genes. The year 2009 saw the first description of NDM-1, a metallo-beta-lactamase. NDM-1 hydrolyzes carbapenems and is not targeted by beta-lactamase inhibitors^22^, thus allowing bacteria expressing NDM-1 to bypass the two main treatment options for ESBL-positive strains. The other known enzymes in *K. pneumoniae* conferring resistance against beta-lactamase inhibitors are *ampC*-type beta-lactamases and inhibitor-resistant beta-lactamases.

There is currently little understanding of why some *K. pneumoniae* sequence types are more successful than others. High-risk lineages in related pathogens, e.g. *Escherichia coli* ST131 and *Salmonella enterica* serovar Typhimurium ST313, acquired multiple antibiotic resistance determinants prior to their clonal expansions. Here, we describe the population structure and resistance profiles of *K. pneumoniae* isolated from a large hospital in Pakistan during routine sampling in 2010–2012. Our analysis reveals that there are many lineages that were prevalent at the time which have subsequently not spread more globally^11^, and that dominant lineages which are now recognised as high-risk clones did not carry NDM-1. We combined short-read and long-read sequencing and phenotypic resistance profiles for selected isolates, and observed NDM-1 to be unstable in some of these *K. pneumoniae* lineages. Our study again strongly emphazises the relevance of the genetic background and intrinsic resistance mechanisms to provide some strains with a competitive advantage within a pool of highly resistant *K. pneumoniae* population.

## Results

### Conserved ESBL gene repertoire vs. high diversity in additional beta-lacatamse genes

The isolates were collected between 2010–2012 through the routine microbiological screening of bacterial infections in The Children’s Hospital, Lahore, Pakistan, and were pre-selected for ESBL expression through the E-test^23^. At this time, ESBL-resistant *K. pneumoniae* were responsible for a significant clinical burden at Lahore hospital. The patients ranged in age between neonates (<29 d) and 15 years, and all received at least one invasive procedure during their hospital stay (97% intravenous lines) as described in detail in Ejaz *et al*.^23^. ESBL-positive *K. pneumoniae* were responsible for a high number of negative outcomes (‘left against medical advice’ or death) in the hospital during the study period (87/214). Whilst the patient symptoms and the overall epidemiology of the strain collection was discussed in detail in Ejaz *et al*.^23^, we now perform a detailed genomic analysis to identify resistance and virulence genes, and explore whether any of these genes conferred a competitive advantage within this highly drug-resistant isolate collection.

**Figure 1:**
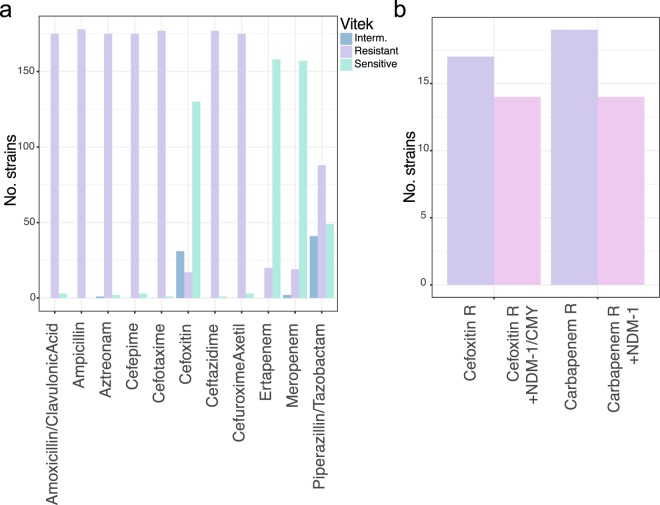
Resistance profiles. (**a**) Vitek data showing the number of strains read as sensitive, intermediate or resistant against the respective antimicrobial. (**b**) Isolates resistant against cefoxitin or carbapenems were assessed for the presence of genes expected to confer this resistance, highlighting a small gap of strains with non-predicted resistance mechanisms.

Phenotypic screening of antibiotic susceptibility (Table S1) identified isolates that were negative for AmpC, but resistant to cefoxitin, a second-generation cephalosporin that is, contrary to other cephalosporins, resistant to the activity of ESBL enzymes^24^ (Fig. 1). To identify any lineages responsible for this resistance, and to assess whether a new resistance mechanism was spreading, we conducted a detailed analysis of the resistance profiles. We combined phenotypic analysis using the Vitek system, which facilitates measuring susceptibility to a wide range of antimicrobials at high throughput, with genomic analysis of candidate acquired resistance genes.

**Figure 2:**
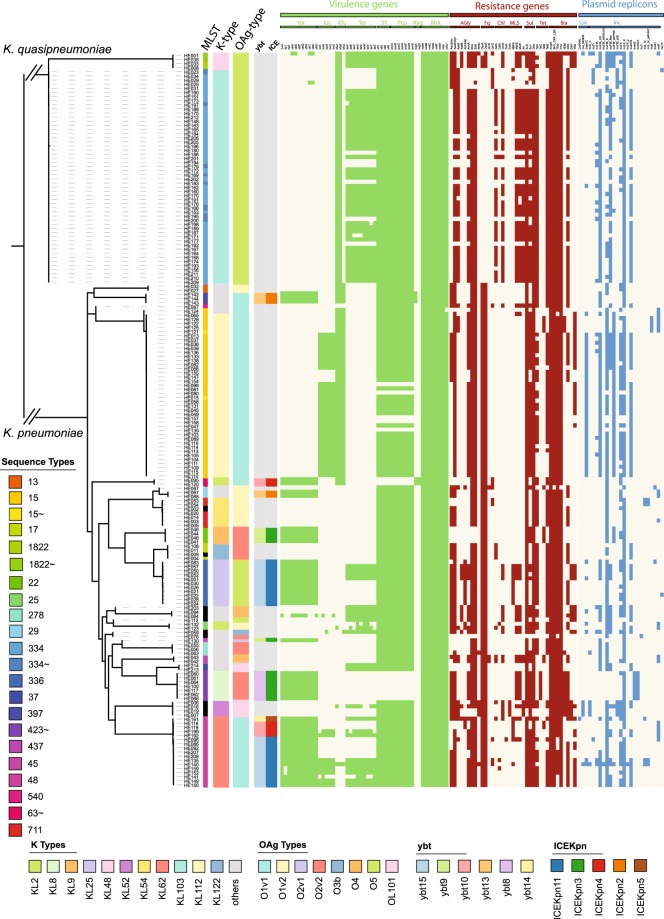
Antimicrobial resistance (AMR), plasmid replicons and virulence genes, and K- and O-type. The guide tree is based on roary, the branches leading to *K. pneumoniae* and *K. quasipneumoniae* are not to scale to facilitate visualisation; and shows the diversity of capsule and O-antigen type, which correlate with sequence types (one sequence type usually shares the capsule- and O-antigen combination). The predictions of AMR genes, virulence genes and plasmid replicons was performed using ariba^43^, a mapping-based approach independent of assemblies. The guide tree is based on roary as in Fig. 1 where the branches leading to *K. pneumoniae* and *K. quasipneumoniae* are not to scale to facilitate visualisation.

*Klebsiella* spp. normally harbour two chromosomally integrated enzymes related to low-level beta-lactam resistance. AmpH is an AmpC-related enzyme functioning as penicillin-binding protein, whereas beta-lactamases of the SHV, OKP or LEN family are usually present as one chromosomal copy in *K. pneumoniae*, *quasipneumoniae* and *variicola*, respectively^25^ (Figs 2 and S1). In addition to these well established resistance genes, we detected a high number of acquired resistance genes, where the extended-spectrum beta-lactamase CTX-M-15 was ubiquituously present. We detected three OXA and three TEM beta-lactamase sequence variants (the TEM DNA sequence variants translate into identical amino acid sequences), and observed that a small number of isolates carried the rare VEB-5 beta lactamase. We furthermore detect several isolates with an additional copy of the SHV beta-lactamase family, most likely acquired on a plasmid. We detected other beta-lactam resistance genes with extended spectrums including the the AmpC enzymes CMY-2 and CMY-6, and the NDM-1 carbapenemases (Figs 2 and S1). The measured MIC values for widely-used antimicrobials of this isolate collection generally matched the genomically-predicted resistances (Table S1, Fig. S1). Intrinsic resistance mechanisms such as deactivation of porins, upregulation of efflux pumps, or differential expression of resistance genes, might account for resistance in the non-matching isolates^26,27^.

Sensitivity was observed among a high number of isolates for cefoxitin and piperacillin-tazobactam. This is expected as cefoxitin is generally insensitive to ESBL^24,28^ (e.g. *bla-CTX-M-15*, which was present in all but one of our isolates), whereas resistance against piperacillin is widespread; in contrast, the beta-lactamase inhibitor tazobactam was still usually effective^24,29^. In most of our isolates, resistance against cefoxitin was explained either by the presence of the carbapenemase *bla-NDM-1* which hydrolyses cefoxitin^30^, the *bla-CMY* AmpC^31^, or both (Fig. S1). Importantly, these data reveal that there was not simply a single acquisition of an additional resistance gene in the population leading to the extended resistance phenotype. Three isolates (HE021, HE205, HE206; Fig. S1) expressed phenotypic resistance that could not be explained by acquired genes. A pan-genome analysis of the *K. quasipneumoniae* lineage reveals a number of differences which might affect drug sensitivity such as transposases and hypothetical proteins (likely phage-derived following comparisons to public databases), however the only clear genetic difference between HE205 and HE206, and closely related sensitive isolates, is a predicted iron uptake system (*fec*) that was absent from the resistant strains. However, it is unclear whether *fec* has any effect on the observed resistance, or whether other changes like differential expression of genes, for example upregulation of efflux pumps or downregulation of porins, leads to cefoxitin resistance in these strains.

In considering other alternative drug treatments for ESBL-positive organisms, we noted that sensitivity against piperacillin/tazobactam (TZA) often coincided with lower MIC values against amoxicillin/clavulanate (Fig. S1; Table S1). This is despite the fact that, based on a phenotypic sensitive/resistant evaluation, these isolates grew at drug levels above the defined resistance threshold, and thus were assessed as amoxicillin/clavulanate resistant. More detailed analysis of the encoded SHV/OKP *bla* genes revealed that several *K. quasipneumoniae* isolates (HE031, HE029, HE025, HE034, HE023; ST334) harboured a *bla-SHV* gene in addition to the intrinsic *bla-OKP*. The hypothesis that this mismatch in genotype and phenotypic resistance was plasmid-derived is supported by the plasmid replicon profiles of these isolates, which differ from closely related strains lacking the additional *bla-SHV* (Fig. 2). These putatively plasmid-borne *blaSHV* genes encode the mutations G238S and E240K, which correlate with TZA resistance or TZA-intermediate sensitivity (Fig. S1)^32–34^. We also observed several sequences encoding the L35Q mutation. However, the L35Q mutation only provides a subtle increase in TZA MICs^32,34,35^, and no mutation was found at S130^36^. In summary, we found four different mechanisms that confer resistance against cefoxitin and/or inhibitor combinations in addition to the endogenous ESBLs. These include NDM-1, CMY-2, CMY-6, and additional SHV copies and their combinations, as well as three strains with unexplained profiles, highlighting the diversity of resistance mechanisms present in our collection.

### No single factor gives competitive advantage

**Figure 3:**
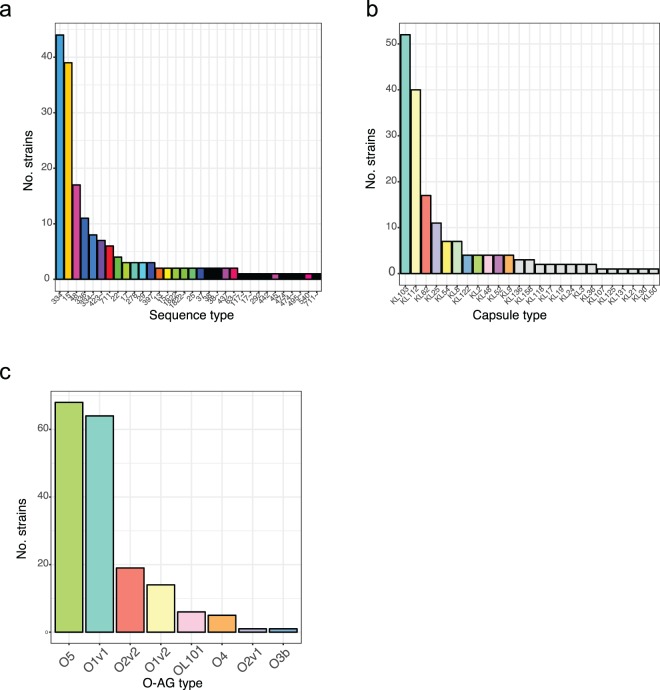
LPS O-antigen, capsule and sequence types in this study. (**a**) Two main (ST15, ST334) sequence types dominate the collection, followed by several medium-abundance (e.g. ST45, ST336) types and a high diversity of low-abundance sequence types. (**b**) The capsule types almost share almost exactly the same level as diversity as the sequence types, with a different capsule type associated with sequence types; whereas the O-antigen types (**c**) are as expected less diverse, but dominated through the commonly as less diverse perceived type O5.

To test whether the observed resistance patterns resulted from expansion of single lineages that had earlier acquired the respective mechanism, or multiple introductions into the same or distinct lineages, we analysed the data in context of their phylogenetic history. Our dataset included 21 different sequence types (STs)^23^ (Fig. 3a), with 56 isolates of *K. quasipneumoniae*, a species that was until recently thought to be less virulent than its related species, *K. pneumoniae sensu stricto* and *K. variicola* (Fig. 3)^23^. The prevalence for each ST ranged from a single to 52 isolates (ST 334), showing that the STs that acquired ESBL genes were not equally competitive in the hospital. These observations suggest that unresolved factors in the genetic background lead to higher prevalence of some lineages over others when all the isolates with a similar resistance profile are evaluated in the same setting.

The capsule (K) is an essential virulence factor for *K. pneumoniae* and, together with lipopolysaccharide O-antigen (O) types, used for typing. K- and O-type can be identified and assigned based on their respective operons in the genomes. Recent work^37,38^ highlighted the considerable diversity found among different *K. pneumoniae* capsule types^39^. This phenomenon is also reflected in our collection, where we observed a different capsule type for almost each sequence type, and no single type correlating with more dominant lineages (Fig. 3b, Table S2). This argues against any one capsule type itself conferring a competitive advantage in this setting, but rather that either multiple capsule types are equally advantageous, and/or that some combinations of capsule type and genotype are more competitive in a clinical setting that others.

In contrast to capsule loci, there was less diversity of LPS O-antigens with two main types in these ESBL producing isolates (Fig. 3c). While other global studies^37,40^ observed a combination of O1 (52%/26%; from Follador *et al*., and Pennini *et al*., respectively), O2 (16%/36%) and O3 (15%/13%) as main LPS types^37,40,41^, we observed O1 (44%) and O5 (38%; vs. 6%/1% in the global studies) as the most common. The high prevalence of O5 was due to a single lineage expansion, one of the two very dominant lineages was O5 (ST334), whereas the other one was O1v1 (ST15). The differences in O-antigen abundance of different studies are highly relevant as O-antigens are often considered vaccine targets in *K. pneumoniae*.

### Virulence factor distribution

The genes encoding the siderophores aerolysin and yersiniabactin were only detected in *K. pneumoniae* isolates, in accordance with a previous large-scale study investigating 2499 isolates^42^ which also reports their absence in *K. quasipneumoniae*. Forty-one isolates, representing 33.6% *K. pneumoniae*, carried the yersiniabactin determinant, and 42 *K. pneumoniae* (34.4%) carried aerobactin. Only 7 of these isolates (5.7%) encoded both siderophores^42^ (Fig. 2), and no salmochelin or colibactin encoding genes were identified.

The profiles present in our strains include six different types of yersiniabactin using the classification of yersiniabactin (ybt) and their associated mobile elements (Kp integrative conjugative element ICEKp) by Lam *et al*.^42^. These ybt loci were present in eight sequence types. Our collection also included a less common combination of ybt8 with ICEKp3 (ST423). In addition, we found one sequence type (ST48) with three different ybt-ICE variants (Fig. 2; ICEKpn11 + ybt15, ICEKpn4 + ybt10, ICEKpn5 + ybt14), suggesting independent acquisition of different siderophore types even within a confined setting.

We also noted a high number of genes predicted as pseudogenes by ariba^43^, especially multiple occurrences of the virulence gene *rmpA2* as pseudogene, which is one of the capsule upregulators and diagnostic for hypervirulent *K. pneumoniae* (Fig. S2a). The *rmpA2* genes were all disrupted through a single-nucleotide addition or deletion in either a poly-G or poly-A region in this gene causing a frameshift as described previously^44^ (Fig. S2a), and the encoded potential for random slip-straind misrepair to bring rmpA2 into frame and increase capsule production. RmpA and aerobactin are indicative of the presence of the *K. pneumoniae* virulence plasmid found in hypervirulent strains^45–48^, and we therefore compared the typical *K. pneumoniae* virulence plasmids, indicative in particular of the hypervirulent clonal group 23 (CG23), with our data. We performed comparisons against the plasmid pSGH10, which is representative of the hypervirulent clade CG23^48^. This comparison showed that sequences similar to the pSGH10 plasmid are present in two main STs in our collection but that both STs lack salmochelin and other elements of the plasmid pSGH10 (Fig. S2b)^49,50^. There was also variability of which regions of the plasmid were conserved within the STs in our study, again highlighting the highly dynamic and plastic nature of *K. pneumoniae* genomes.

### The plasmid background of NDM-1

To identify the number of different plasmids that were present in our dataset, and to assess how many are still in circulation (e.g. reported in the recent literature), we performed long-read sequencing of representative isolates, using Pacific Biosciences (PacBio) technology (Tables 1, S2). Some NDM-1 genes detected in Illumina reads were not confirmed by PacBio sequencing, and were only detected with low read coverage (Table 1 and Fig. S3). Hence, we performed additional MIC tests for meropenem (E-tests, see methods; Table 1) using cultures generated after DNA extraction for sequencing. This phenotypic analysis confirmed that, where only lower read coverage for NDM-1 was observed in the earlier Illumina data, the bacteria were meropenem-sensitive indicating either loss of the plasmid carrying NDM-1 or of a highly mobile cassette carrying NDM-1. Even though we cannot exclude contamination during the multiplexed short-read sequencing run or the earlier stages of the sample preparation, the unstable nature of NDM-1 has been observed previously in several independent reports, where loss can occur after two generations of laboratory subculture, and even under meropenem selection^51–53^. The putative loss of the entire plasmid carrying NDM-1 in part of our samples, or at least a larger transposable cassette, is further supported by the observation that several resistance genes that were predicted at equally low abundance in Illumina sequencing, were absent from the PacBio assemblies, indicating an unstable plasmid carrying these resistances, which included NDM-1 (Fig. S3).

**Table 1:** Detection of carbapenemase using minimum inhibitory concentration and gene prediction (ariba read coverage).

Strain	MIC (Meropenem)	NDM-1 coverage
HE006 (14893_8#74)	64	75.0
HE007 (14893_8#75)	32	83.8
HE021 (14893_8#89)	2	Not Detected
HE016 (14893_8#84)	64	59.3
HE019 (14893_8#87)	64	79.1
HE121 (14936_2#2)	32	105.4
HE122 (14936_2#3)	16	49.4
HE065 (14936_3#41)	<1.25	Not Detected
HE125 (14936_2#6)	32	73.3
HE128 (14936_2#9)	32	43.4
HE001 (14893_8#69)	16	8.1
HE035 (14936_3#11)	4	Not Detected
HE063 (14936_3#39)	16	6.2
HE055 (14936_3#31)	16	Not Detected
HE060 (14936_3#36)	64	44.7
HE061 (14936_3#37)	64	47.1
HE062 (14936_3#38)	64	53.8
HE213 (14936_2#94)	<1.25	Not Detected
HE064 (14936_3#40)	64	52.9
HE066 (14936_3#42)	64	56.3
HE100 (14936_3#76)	64	43.5
HE117 (14936_3#93)	64	53.9

**Figure 4:**
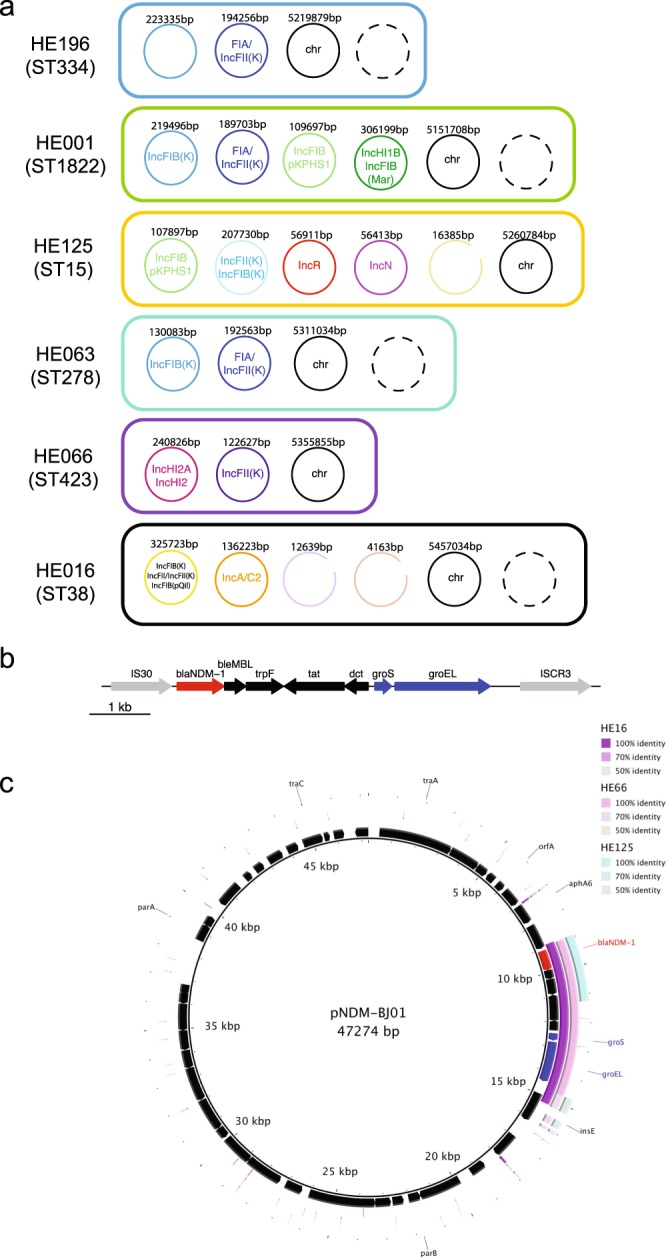
Complete plasmid repertoire of selected isolates. (**a**) The plasmids, their replicons and sizes as predicted after pacbio sequence analysis are shown for the respective isolates. (**b**) A close-up of the original NDM-1 cassette^96^. (**c**) The three plasmid contigs from the PacBio assemblies of HE016, HE066 and HE125 which contain the NDM-1 gene, were compared against a plasmid encoding the original cassette as described for *Acinetobacter*^55^. This shows the different levels of conservation of the cassette, whilst nothing else of the original plasmid can be found.

Incompatibility group F and H plasmids were fixed in the population across all sequence types, with IncN and IncR only rarely observed; this is in agreement with a large comparative study describing IncFII, IncA/C, IncL/M, and IncI1 as the main resistance-carrying Inc types in *Klebsiella*^54^ (Figs 2 and 4a). Two of the plasmids resolved by long-read sequencing (strains HE016 and HE066) show the original, more complete cassette including the GroEL/S from the *Acinetobacter* construct^55^ (Fig. 4b,c), whilst in one case (HE125) a smaller portion of this gene region is conserved (Fig. 4c); this variation in the different genes surrounding NDM-1 has been described in other studies^55–58^.

### Comparison of ST15 isolates from Pakistan with a reported ST15 outbreak in Nepal

**Figure 5:**
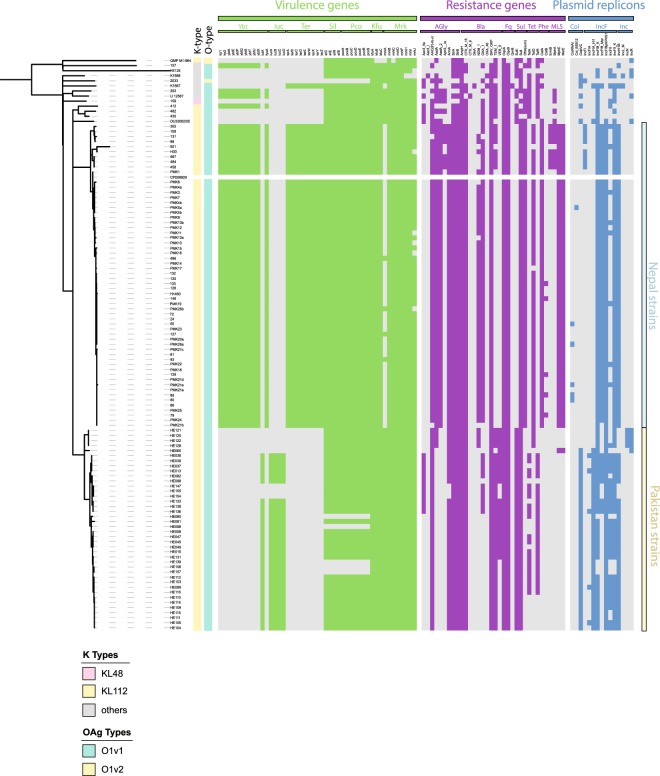
Comparison of the ST15 isolates from an outbreak in Nepal and Pakistan. Plasmid replicons, resistance genes and virulence genes were predicted by ariba; the tree is a whole-genome tree after removing recombination with gubbins against the reference strain from Nepal (CP008929.1).

ST15 is an example of a widespread problematic *K. pneumoniae* clade. Given the relative geographic proximity between Pakistan and Nepal, we analysed the relationsips between the two ST15 lineages reported in Nepal^59,60^ with those isolates in our collection (Table S3). We assessed the presence or absence of genes via a pan-genome analysis of a subset of isolates, as well as mapping of the short read data for the same isolates against a PacBio-sequenced reference sequence (accession number CP008929; Figs 5 and S4) from the Nepal outbreak^59^. The phylogeny indicated that the ST15 population from Lahore was comprised of several subgroups, and differences indicated by longer branches correlated with changes seen in the mapping results (Fig. S4). Even within the tight group of the isolates from Lahore, at least three different sublineages had emerged. Each of these sublineages lack one, two or three regions found in the reference strain from Nepal (Fig. S4). A detailed overview of resistance, virulence gene profiles, plasmid replicons and surface determinants (capsule and O-antigen type) is provided in Fig. S4, and includes part of the Type VI secretion system as well as cellulose biosynthesis. Analysing the capsule operons, we observed the same novel capsule type as described in the Nepal isolates^60^ (Fig. 5), further highlighting that ST15 appears to be spreading throughout the South Asian subcontinent.

In contrast, a comparison of the plasmid content showed almost entirely different compositions of plasmids in Nepal and Lahore, Pakistan (Figs 5 and S5). Only one of the Nepal plasmids (plasmid A) is partially conserved in the isolates we examined (Fig. S5), in accordance with the absence of NDM-1 (Fig. S5). Several gene clusters from the Nepal outbreak plasmids are conserved and likely to be encoded on different plasmids, including (Nepal plasmid A) *lacIZY*, phosphate transport (*phnD*/*E ptxD*); arsenic (*ars*), copper (*pco*), silver (*sil*) and cation (*cus*) efflux systems, as well as plasmid maintenance proteins from this plasmid. Only sparse similarity can be found with other plasmids (e.g. Nepal plasmid C streptomycin resistance), in accordance with the different virulence and resistance profiles of the two hospital lineages. A subgroup of the ST15 isolates from Pakistan (HE080, HE098, HE139, HE157, HE158) did not share the genes from plasmid A in Nepal, which can also be seen by their different virulence factor profile (Figs 5, S4 and S5).

## Discussion

The *K. pneumoniae* isolates described in this study represent routinely collected samples in a Pakistan hospital of the *K. pneumoniae* species complex over an extended period^23^. The main burden of global clinical isolates in hospital strains is usually associated with *K. pneumoniae sensu stricto*. There were two highly prevalent STs, and one of these was *K. quasipneumoniae* subsp. *similipneumoniae*, a subspecies that was previously thought to be less clinically important (Fig. 2)^23^. The major *K. quasipneumoniae* sequence type (ST334) has been rarely observed in other studies (e.g. 1/250^61^; 1/167^62^; 1/328^63^; 2/198^64^), but was common in Lahore – we found 52 isolates (44 if excluding ST variants) all originating from bloodstream infections, representing 29.2% (24.7%) of samples investigated. There is however very little data on *K. pneumoniae* diversity from the region, and it is unclear whether the expansion of ST334 was an unusual occurrence, or whether this ST is still dominant in the area. Given its similarity to *K. pneumoniae* in standard diagnostics, *K. quasipneumoniae* numbers are very likely underestimated in routine testing^65^.

With the continuous loss of effective antimicrobials and the lag in the development of new ones being released, antibody-based treatments should be considered and have support through governments and global health programs^66,67^. There are challenges associated with immunoprophylaxis against *Klebsiella* infections as there is no single prevalent capsule- or O-antigen type. Two large-scale analyses of global datasets including all three species identified serotypes O1, O2 and O3 in 80%/75% of hospital infections^37,40^. We observed a high number of non-O1/O2 types in our collection, and the increase of genomic and functional studies of O-types in the past years led to an increase in recognised LPS diversity. The diversity and distribution is of high importance as O-antigen has often been investigated as a potential target for an anti-*Klebsiella* vaccine, as only few O-antigen serotypes are known, in contrast to the considerable diversity in capsule types^37,38^. However, the lack of apparent correlation to disease severity^37^ and LPS type indicate that if an O-antigen targeted vaccine were introduced, switches to non-vaccine targeted LPS serotypes or the emergence of new serotypes might occur once immune pressure is applied^68,69^.

Given the reported association of the siderophore yersiniabactin with disease, we tested whether a correlation existed between siderophores and patient outcome, but neither yersiniabactin nor aerobactin were significantly associated with negative or positive events (death or discharged), in contrast to a recent description of high mortality with a reduced virulence factor repertoire of ST11^70^ (details Table 2). We observed a similar prevalence of aerobactin and yersiniabactin in our population, and neither of these was associated with higher number of isolates which could indicate competitive advantage. Both were present only in *K. pneumoniae*, and relatively few strains (7; 5.7%) encoded both siderophores. This analysis, showing parts of the virulence plasmid as well as high levels of resistance in the same strains, suggests that the evolutionary convergence between virulent and resistant strains was already occurring within these historical isolates^63^. Other studies have also suggested that the accumulation of virulence factors is an ongoing process in *K. pneumoniae*, and more work needs to be done in longitudinal studies to monitor the changes of the virulence potential of sequence types over time^71^. This is particularly relevant given recent observations of high mortality associated with strains with reduced virulence potential^70^.

**Table 2:** Testing outcome versus presence of yersiniabactin or aerobactin.

Siderophore	Outcome	Discharged	Total
	Death		
Yersiniabactin+	16	26	42
Yersiniabactin−	34	75	109
Total	50	101	151
p-value^a^	*p*_*chi*_ = *0.5388*		
Aerobactin+	13	22	35
Aerobactin−	37	79	116
Total	50	101	151
p-value^a^	*p*_*chi*_ = *0.709*		

^a^P-value determined via a chi-square test of independence using the R function chisq.test(input matrix).

The collection is derived from a time (2010 to 2012) where carbapenem resistance was still at comparatively low levels and NDM-1 had only recently been introduced into the *K. pneumoniae* population, whereas now we see very high prevalence in LMIC as well as high-income countries^2,26,27^. Even though the KPC carbapenemase enzyme was described first in 1996 in the USA^72^, it spread comparatively slowly but has become highly problematic in the American continent and southeast Asia (China, Taiwan, Singapore)^56^. NDM-1 was first reported in 2008^22^, but spread rapidly within the Indian subcontinent, and across all continents by 2010^73,74^. Our dataset contains several lineages with NDM-1, however these did not become globally spread high-risk lineages in the subsequent years. Acquisition of an NDM-1 plasmid *per se* is neither a sign that the plasmid will spread stably with the population, nor that this lineage has a major advantage over all carbapenemase-negative lineages, which likely reflects intrinsic factors that need to be present to provide an NDM-1 positive lineage a competitive advantage over other ESBL-positive lineages as observed in Nepal. The apparent instability of the NDM-1 locus in the isolates we examined, most likely through plasmid instability, suggests that laboratory analysis of this important resistance must be conducted carefully.

Monitoring resistance to inhibitor-based treatments is crucial as this combination treatment is a common clinical response to ESBL infections^29^, and there are ongoing efforts to develop inhibitors against beta-lactamases and more recently also carbapenemases^75–77^. There is considerable subtlety in the evolution of drug resistance, and especially for beta-lactams a variety of activity spectra across resistance enzymes in one strain is likely to be of importance, as enzymes with lower activity spectra are often more resistant to inhibitors than broad-spectrum ESBL enzymes^78^. Detailed analyses, including the fitness cost of resistance genes as indicated by the unstable nature of NDM-1 or, as contrasting example, the rapid spread and fixation of CTX-M-15, are crucial to our understanding of the different mechanisms of resistance and their fixation in a population.

## Materials and Methods

### Strain collection

The strains were derived from routine diagnostic bacterial cultures in the Microbiology Department of The Children’s Hospital & The ICH Lahore, Lahore, Pakistan, during 2010–2012, where 13% of all laboratory-positive samples were *K. pneumoniae*. The strains were pre-selected for ESBL resistance, and a detailed description of the infections in the hospital during the time as well as specific data on patient symptoms and other metadata regarding the strains analysed here can be found in Ejaz *et al*.^23^. The study was ethically approved by the institutional review board of The Children’s Hospital, Lahore under No. 1625/PH&I. The studies were compliant with the ethics approval granted by the Institutional Review Board of the Lahore Children’s Hospital, all the methods were performed in accordance with the standard ethical guidelines and do not include any human or animal trials.

### PacBio DNA preparation and sequencing

DNA from overnight cultures was isolated by Phenol:Chloroform:Isoamyl Alcohol (25:24:1) and Chloroform:IAA (24:1) extractions using Phase-lock tubes (Qiagen) and re-dissolved in 10 mM Tris pH 7.4 buffer. Sequencing was performed on the PacBio RSII using P6/C4 sequencing chemistry, the library was prepared using the SMRTbell Template Prep Kit 1.0. Filtered sub-reads were generated with the pacbi-smrt software, and assembled with canu v1.1^79^. The assemblies were then circularised using circulator v1.5.3^80^ with canu as assembler, and polished with unicycler-polish v0.4.0^81^ and the Illumina reads of the respective sample. HE016 gave better results in assembling with the unicycler-hybrid assembler v0.4.0 combining the PacBio data with the Illumina reads^81^, and was subsequently circularised and polished as above. All assemblies were then annotated with PROKKA v1.11^82^.

### Bacteria Mapping and Variant Detection

Mapping was performed against the chromosome of *Klebsiella pneumoniae* NTUH-K2044 (AP006725), a published whole genome from an outbreak in Nepal^59^ and HE196 (this study). Sequence reads were mapped against the reference genome as indicated using SMALT^83^ (v0.7.4) to produce a BAM file. Variation detection was performed using samtools mpileup v0.1.19 bcftools v0.1.19 to produce a BCF file of all variant sites.

### Pan-genome analysis

The samples as indicated in the respective experiments were assembled and annotated as described above, and the GFF3 files generated by PROKKA v1.11^82^ were used as input for the pan-genome pipeline Roary^50^ (v3.7.0) with a BLASTp percentage identity of 90% and a core definition of 99%. This gives a core gene alignment of 3486 genes for all strains from this study (Figs 1, 2). The core gene alignment was generated with mafft v7.205^84^; SNPs were first extracted using snp-sites v2.3.2^85^, and then a maximum likelihood tree with RAxML v8.2.8^86^ was calculated. For the ST15 comparison, the pan-genome was calculated as described above, and gene presence/absence analysed further using R v3.4.2 and the resulting presence/absence matrix and phandango^87^.

### Phylogenetic analyses

Trees were calculated using RAxML^86^ (v8.2.8) with the time-reversible GTR model and 100 bootstrap repeats. Tree demonstrations were prepared in itol^88^, coverage plots were generated using bedtools genome coverage^89^ and displayed using the R package ggplot v2.2.1^90^. For whole-genome mapping trees, recombinant regions were removed using Gubbins^91^ (v1.4.9) and a maximum likelihood tree calculated with RAxML to obtain bootstrap support values, as described above, and sites with more than 5% N were not considered in the tree calculation.

### Gene content analysis

For the determination of the antibiotic resistance profile, the virulence factor profile and plasmid types, we used ariba. The resistance, virulence and plasmid profiles were matched against the modified version of ARG-ANNOT^92^ available at the SRST2 website (https://github.com/katholt/srst2/tree/master/data; download date 02.10.2016), a dataset of virulence factors obtained from the *Klebsiella*-specific BIGSDB (http://bigsdb.pasteur.fr/klebsiella/klebsiella.html; download date 22. 02. 2016), and the PlasmidFinder database as implemented in ariba v2.10.0^93,43^. Sequence gene profiles and types were determined using MLST check^94^ comparing assembled genomes against the MLST database for *Klebsiella pneumoniae* (pubmlst.org/Klebsiellapneumoniae/). For further resistance determinants (SNP- based or porin inactivation), a database with the genes of interest was created as outlined in the ariba manual pages. The assignment of *bla-SHV* alleles was controlled based on amino acids using the beta-lactamase database http://www.laced.uni-stuttgart.de. Yersiniabactin and ICE alleles were annotated using kleborate^42^ (https://github.com/katholt/Kleborate). Capsule and LPS O-antigen was annotated using kleborate and a custom database of LPS O-antigens^37^. The assignment to O3 subgroups was updated based on recent nomenclature (O3l/s; now a/b/c) using the online tool Kaptive^41^ (http://kaptive.holtlab.net/).

### Minimum inhibitory concentrations (MIC) measurements

All isolates were analysed using the VITEK 2 system (bioMérieux, UK). In brief, suspensions of colonies were made in 0.45% saline solution from growth on iso-sensitest agar (Thermo Scientific Oxiod, UK), adjusted to a turbidity equivalent to that of a 0.5 McFarland standard and used to load the test cards, using manufacturer’s instructions. The *Enterobacteriaceae* AST-N350 cards was automatically filled, sealed and inserted into the VITEK 2 reader–incubator module (incubation temperature 37 °C), and fluorescence measurements were performed every 15 min for up to 18 h. For detailed analysis of several NDM-1 containing strains, the MICs for meropenem were also assessed manually following the protocol of Wiegand 2008^95^ (Table 1).

## Supplementary information

Figures S1-S5

Table S1-S3

## Data Availability

Details on strains and accession numbers can be found in Table S1 (Vitek resistance data), Table S2 (accessions and further information on isolates from Pakistan) and Table S3 (additional published strains used for the analyses). Data provided for the Pakistan samples includes accessions for raw sequence data as well as assemblies of both Illumina and PacBio sequenced genomes and patient metadata. All relevant data is in the manuscript and supporting material, alignment and tree files are available for download at figshare https://figshare.com/s/cdaddb659a6e178102df.
