# Supplementary material for: Resistance mechanisms and population structure of highly drug resistant Klebsiella in Pakistan during the introduction of the carbapenemase NDM-1
Source: Sci Rep. 2019 Feb 20;9:2392. doi: 10.1038/s41598-019-38943-7 (PMC6382945; doi:10.1038/s41598-019-38943-7)
Supplement: Supplementary file 1 — Figures S1-S5 [file 41598_2019_38943_MOESM1_ESM.pdf]

Resistance mechanisms and population structure of highly drug resistant *Klebsiella* in Pakistan during the introduction of the carbapenemase NDM-1

Eva Heinz[a*]

Hasan Ejaz[b,c,d]

Josefin Bartholdson Scott[e]

Nancy Wang[c]

Shruti Gujaran[c]

Derek Pickard[a]

Jonathan Wilksch[c]

Hanwei Cao[c]

Ikram-ul-Haq[f]

Gordon Dougan[a,e]

Richard A Strugnell[c*]

a: Parasites and Microbes, Wellcome Trust Sanger Institute, Hinxton, CB10 1SA, UK

b: Department of Clinical Laboratory Sciences, CAMS, Jouf University, Al-Jouf, Kingdom of Saudi Arabia

c: Department of Microbiology and Immunology, The University of Melbourne, at Peter Doherty Institute for Infection and Immunity, Melbourne, Australia

d: Department of Microbiology, The Children's Hospital & The Institute of Child Health, Lahore, Pakistan

e: Department of Medicine, University of Cambridge, Cambridge, UK

f: Institute of Industrial Biotechnology, GC University, Lahore, Pakistan

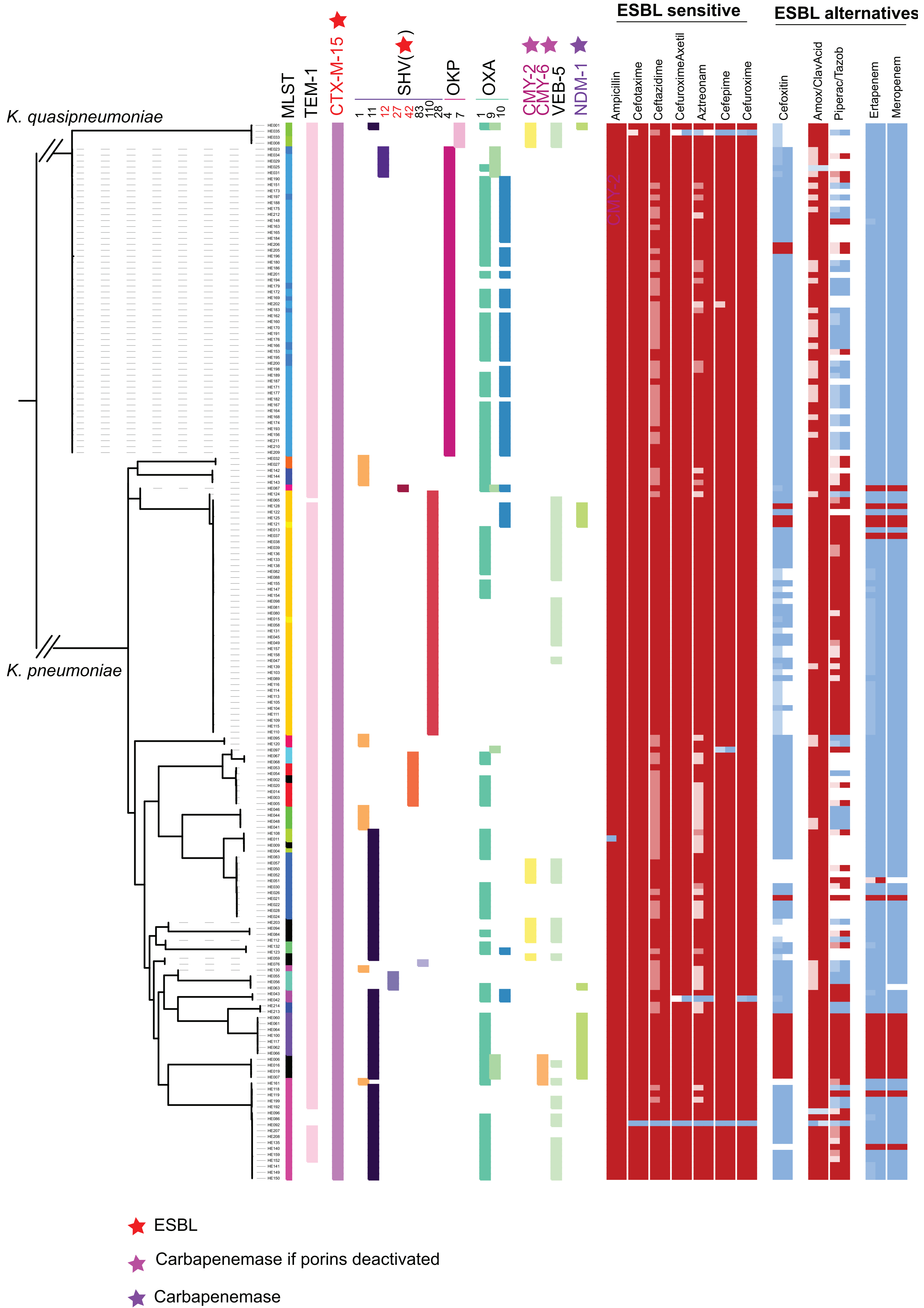

ESBL sensitive
ESBL alternatives
K. quasipneumoniae
K. pneumoniae
MLST
TEM-1
CTX-M-15
SHV( )
1
11
12
27
42
83
110
28
OKP
4
7
OXA
1
9
10
CMY-2
CMY-6
VEB-5
NDM-1
Ampicillin
Cefotaxime
Ceftazidime
CefuroximeAxetil
Aztreonam
Cefepime
Cefuroxime
Cefoxitin
Amox/ClavAcid
Piperac/Tazob
Ertapenem
Meropenem
ESBL
Carbapenemase if porins deactivated
Carbapenemase

Fig. S1: Details of predicted beta-lactamase enzymes and resistance profiles to treatment alternatives for ESBL organisms. The guidance tree is as in Fig. 1. All isolates with TEM or CTX genes encode for the broad-spectrum beta-lactamase TEM-1 and the ESBL enzyme CTX-M-15, respectively, and only the VEB-5 and NDM-1 allele, and two alleles for the AmpC CMY (2 and 6) could be identified. *Bla*-SHV can have different activity spectrums; the red stars indicate extended-spectrum beta-lactamase activity. Pink indicates weak carbapenemase activity which needs additional mutations (e.g. porin deactivation) to confer resistance, dark violet denotes carbapenemase activity. The allele assignment was controlled at the beta-lactamase online database (http://www.laced.uni-stuttgart.de); activity assignments are according to[97]. Vitek measurements are compared with the predicted known resistance-conferring genes for aminoglycosides, fluoroquinolones, sulfonamindes and beta-lactams (including carbapenems). Red indicates resistant, white indicates intermediate, blue indicates sensitive measurement; details are given in Table S2.

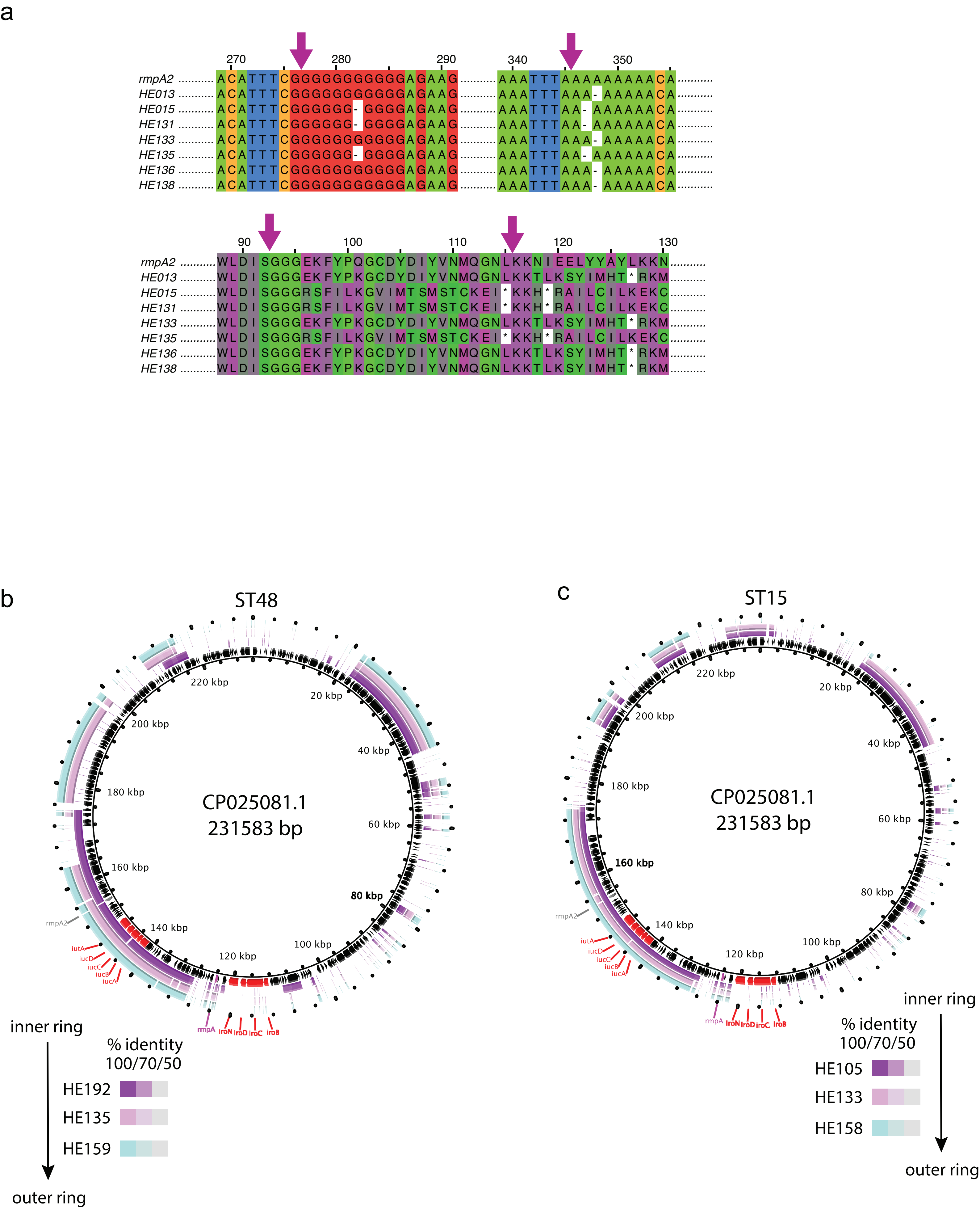
a
270
280
290
340
350
rmpA2
HE013
HE015
HE131
HE133
HE135
HE136
HE138
90
100
110
120
130
rmpA2 WLDISGGGEKFYPQGCDYDIYVNMQGNLKKNIEELYYAYLKKN
HE013 WLDISGGGEKFYPKGCDYDIYVNMQGNLKKTLKSYIMHT*RKM
HE015 WLDISGGGRSFILKGVIMTSMSTCKEI*KKH*RAILCILKEKC
HE131 WLDISGGGRSFILKGVIMTSMSTCKEI*KKH*RAILCILKEKC
HE133 WLDISGGGEKFYPKGCDYDIYVNMQGNLKKTLKSYIMHT*RKM
HE135 WLDISGGGRSFILKGVIMTSMSTCKEI*KKH*RAILCILKEKC
HE136 WLDISGGGEKFYPKGCDYDIYVNMQGNLKKTLKSYIMHT*RKM
HE138 WLDISGGGEKFYPKGCDYDIYVNMQGNLKKTLKSYIMHT*RKM
b
ST48
CP025081.1
231583 bp
20 kbp
40 kbp
60 kbp
80 kbp
100 kbp
120 kbp
140 kbp
160 kbp
180 kbp
200 kbp
220 kbp
rmpA2
iutA
iucD
iucC
iucB
iucA
rmpA
iroN
iroD
iroC
iroB
inner ring
% identity
100/70/50
HE192
HE135
HE159
outer ring
c
ST15
CP025081.1
231583 bp
20 kbp
40 kbp
60 kbp
80 kbp
100 kbp
120 kbp
140 kbp
160 kbp
180 kbp
200 kbp
220 kbp
rmpA2
iutA
iucD
iucC
iucB
iucA
rmpA
iroN
iroD
iroC
iroB
inner ring
% identity
100/70/50
HE105
HE133
HE158
outer ring

Fig. S2: Comparison of isolates with *rmpA2* pseudogenes to Klebsiella virulence plasmids. (a) The sequences retrieved from ariba predicted as *rmpA2* pseudogenes were retrieved and aligned to a reference sequence (amino acids NP_943354.1 and the respective CDS). Two homopolymer regions, a poly-G and a poly-A repeat, show mutations that lead to subsequent frameshifts and stop codons in the translated sequence. The arrows mark the corresponding positions in the amino acid and nucleotide alignments to facilitate orientation. Colours, nucleotides (upper panel); helix propensity (lower panel); as implemented in JalView[98] **(b)** The relevant Illumina contigs were extracted using blastn and abacas, and mapped against the reference plasmid pSGH10[70,48] using BRIG[49] to further illustrate the partial conversation of the virulence plasmid also in the assembled data.

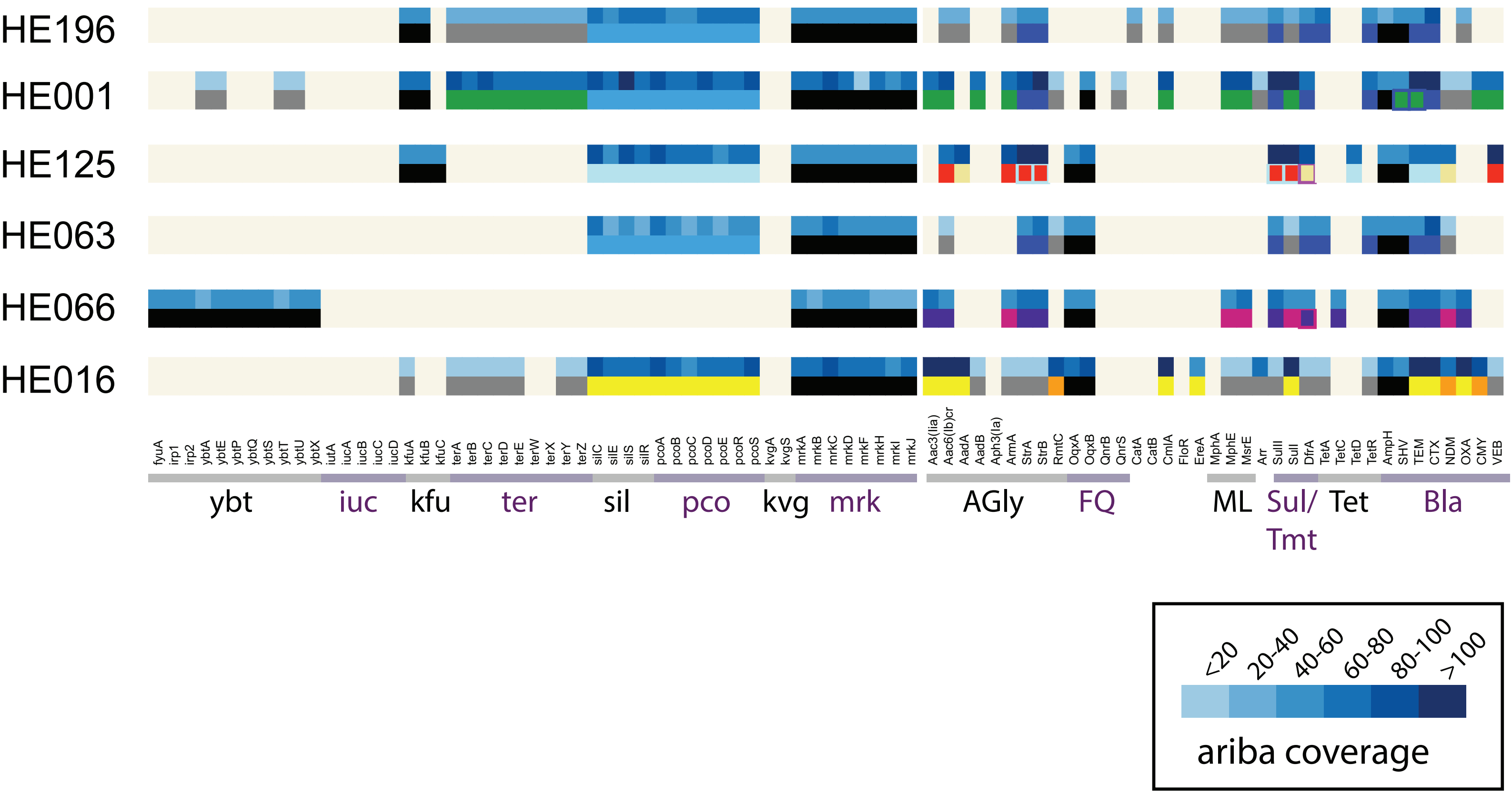

HE196
HE001
HE125
HE063
HE066
HE016
fyuA irp1 irp2 ybtA ybtE ybtP ybtQ ybtS ybtT ybtU ybtX iutA iucA iucB iucC iucD kfuA kfuB kfuC terA terB terC terD terE terW terX terY terZ silC silE silS silR pcoA pcoB pcoC pcoD pcoE pcoR pcoS kvgA kvgS mrkA mrkB mrkC mrkD mrkF mrkH mrkI mrkJ Aac3(IIa) Aac6(Ib)cr AadA AadB Aph3(Ia) ArmA StrA StrB RmtC OqxA OqxB QnrB QnrS CatA CatB CmlA FloR EreA MphA MphE MsrE Arr SulII SulI DfrA TetA TetC TetD TetR AmpH SHV TEM CTX NDM OXA CMY VEB
ybt
iuc
kfu
ter
sil
pco
kvg
mrk
AGly
FQ
ML
Sul/ Tmt
Tet
Bla
<20
20-40
40-60
60-80
80-100
>100
ariba coverage

Fig. S3: The plasmid diversity for representative isolates. The contigs obtained from PacBio for selected strains are shown; plasmid replicons as predicted by the PlasmidFinder web server (https://cge.cbs.dtu.dk/services/PlasmidFinder/[93]. An overview of the resistance genes as predicted by ariba is shown on a colour gradient per strain (upper layer) displaying the read coverage of the hit; and the layer below these indicate on which plasmid the respective genes were found (colours according to the cell overviews, lower panel). Black indicates chromosomal, dark grey indicates gene only found in Illumina reads but not in the PacBio data. The presence of genes was investigated by blastn, only complete perfect (100% identity) hits were considered.

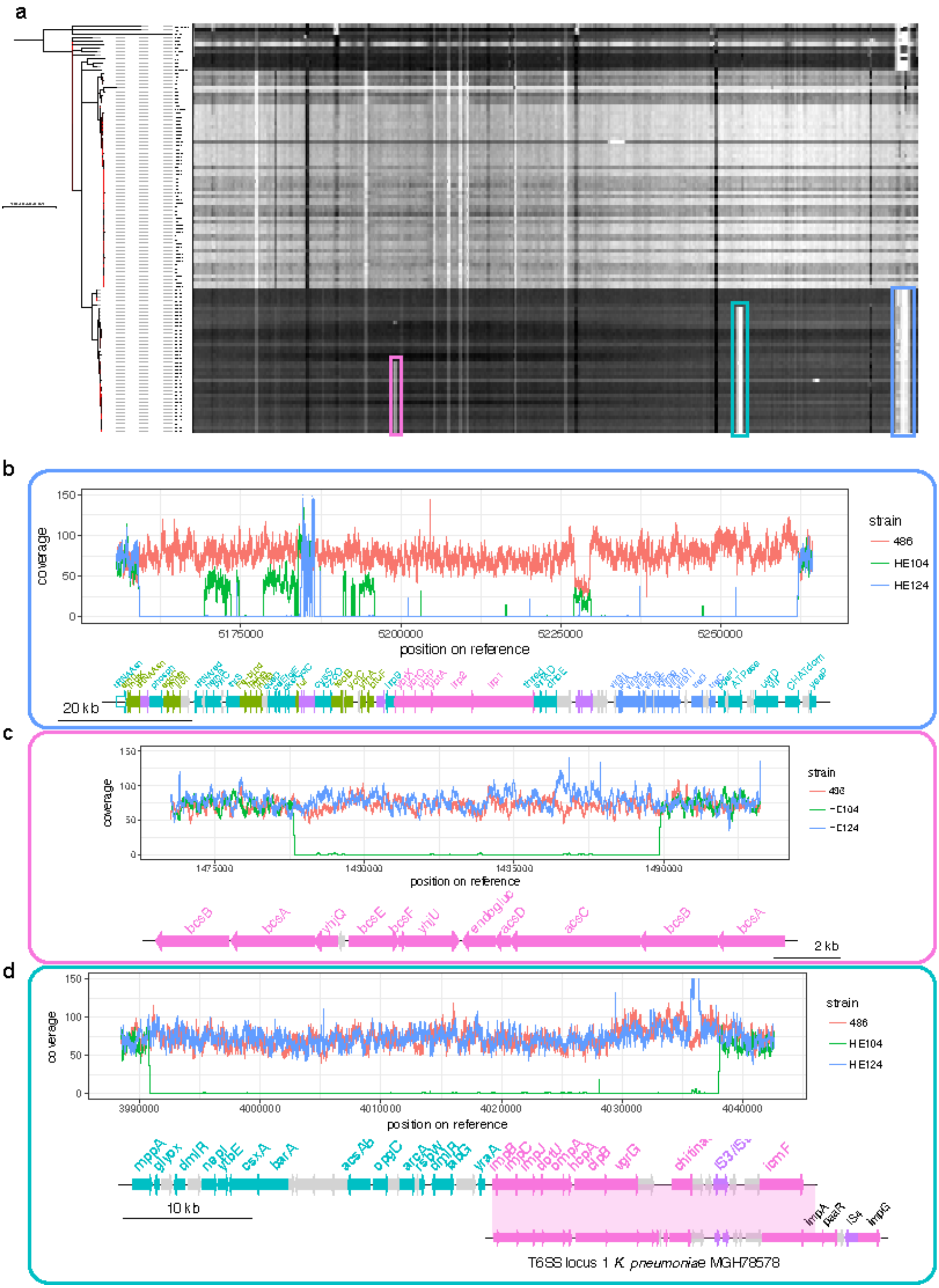
a
b
coverage
position on reference
strain
486
HE104
HE124
20 kb
c
bcsB
bcsA
yhjQ
bcsE
bcsF
yhjU
acsD
acsC
2 kb
d
10 kb
T6SS locus 1 K. pneumoniae MGH78578

Fig. S4: In-depth comparison of expanded lineages. (a) The isolates belonging to ST15 of this study was compared to the ST15 outbreak lineage from Nepal[59,60] by mapping against the completed genome of an isolate from the Nepal outbreak[59], which also carried four plasmids. The heatmap shows the mapping coverage across the chromosome before removal of recombination from white (low coverage) to black (high coverage), emphasizing the large deletions and acquisitions in the Pakistan and Nepal line, respectively. Even coverage of the genome could be observed, with three genetic islands missing in different subsets of our isolates, indicating the dynamic within this closely related lineage. The tree was constructed after removing recombination by gubbins, bootstrap values are depicted as colour gradient; from red (0) to black (100). **(b)** A detailed view of two regions in the genome absent in groups within the isolates from this study (plotted areas: 1473529-1493215, region 1; 3988478-4042608, region 2; coordinates from CP008929.1), but present in the lineage from Nepal, as well as other isolates from the same ST but not part of the clonal lineages as outgroups. In addition, the Nepal lineage acquired the chromosomal locus including yersiniabactin[42]; plotted region 5155581-5264456); these three regions were also removed by gubbins before the tree construction.

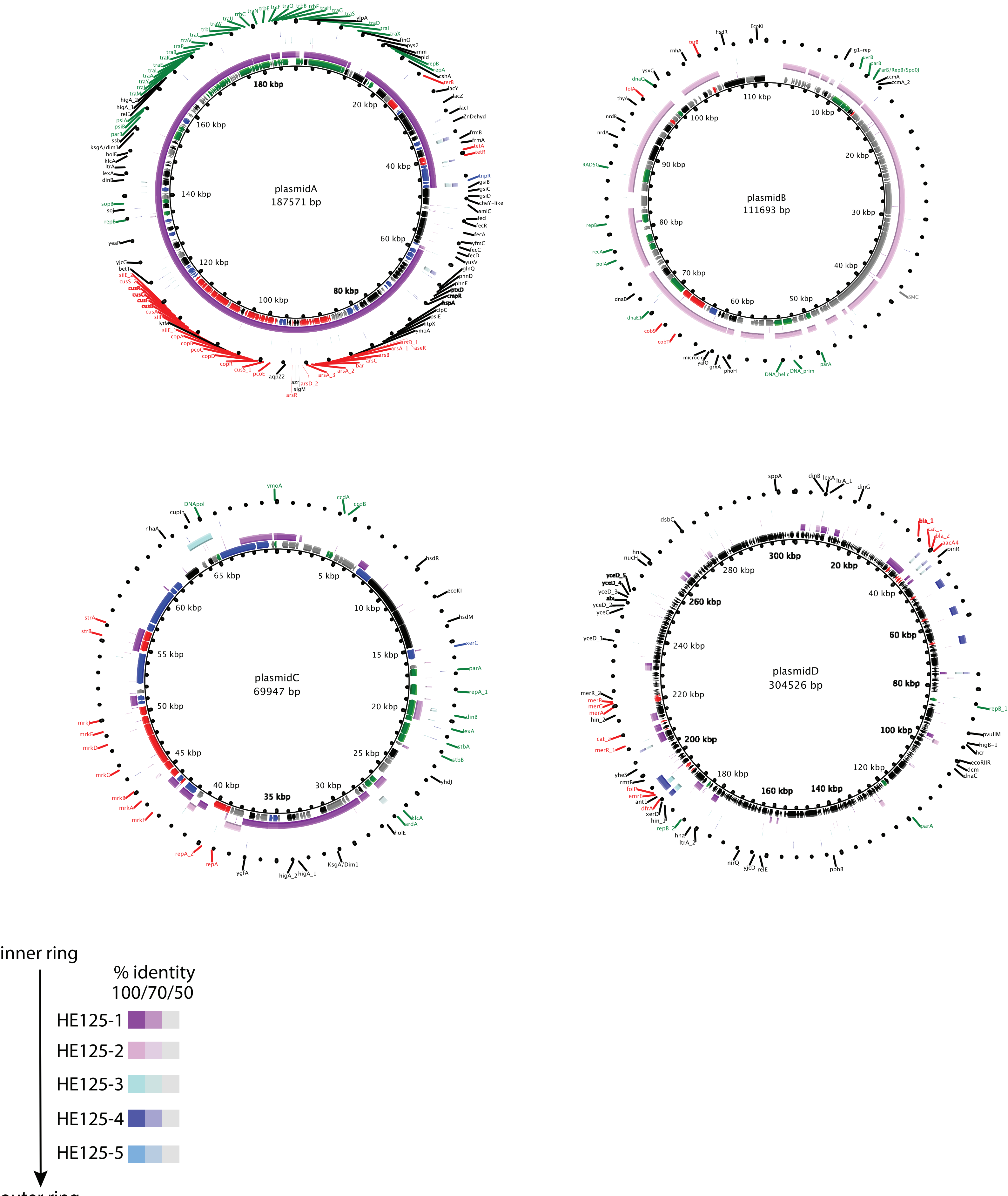
plasmidA
187571 bp
plasmidB
111693 bp
plasmidC
69947 bp
plasmidD
304526 bp
inner ring
outer ring
% identity
100/70/50
HE125-1
HE125-2
HE125-3
HE125-4
HE125-5

Fig. S5: Comparison of the plasmid profiles between the Pakistan and Nepal ST15 lineage. The full genome sequence of a representative strain from the Nepal outbreak[59] was used as reference, and all five plasmid contigs from HE125, a representative of the related ST15 lineage from Nepal, were compared against the four plasmids as annotated (plasmid A = CP008930.1, B = CP008931.1, C = CP008932.1, D = CP008933.1). The legend indicates similarity values for the different plasmid contigs from the HE125 strain.

Table S2: Details on the strains from Pakistan.

Table S1: Vitek measurements.

Table S3: Additional strains included in the detailed analysis of ST15.
